# Intake of 25-Hydroxyvitamin D_3_ May Reduce the Severity of Upper Respiratory Tract Infection: Post hoc Analysis of a Randomized, Double-Blind, Placebo-Controlled, Parallel Group Comparison Study

**DOI:** 10.3390/nu12123769

**Published:** 2020-12-08

**Authors:** Yoshiki Shimizu, Yukihiko Ito, Nobuo Uotsu, Kei Yui

**Affiliations:** FANCL Research Institute, FANCL Corporation, 12-13 Kamishinano, Totuka-ku, Yokohama 244-0806, Kanagawa, Japan; itou@fancl.co.jp (Y.I.); uotsu_nobuo@fancl.co.jp (N.U.); ke-yui@fancl.co.jp (K.Y.)

**Keywords:** 25-hydroxyvitamin D_3_, upper respiratory tract infection, common cold

## Abstract

To evaluate the effects of 25-hydroxyvitamin D_3_ (25OHD) on symptoms at the onset of the upper respiratory tract infection (URTI) in subjects with insufficient or deficient serum 25-hydroxyvitamin D levels, we conducted a post hoc analysis of data from a randomized, placebo-controlled study; the subjects received 10 μg of 25OHD per day or a placebo for 16 weeks. The Wisconsin Upper Respiratory Symptom Survey-21 was used to determine URTI. The study endpoints included WURSS-21 scores, number of URTI events, and proportion of medication (antibiotics, antipyretic analgesics) usage. We found that the physical symptom scores for “Runny nose,” “Sneezing,” and “Head congestion” were significantly lower in the 25OHD group than in the placebo group; for all items except “Breathe easily, “the quality of life” scores were significantly improved in the 25OHD group. There was no significant difference in the number of URTI events or the proportion of medication use between the groups. Collectively, the findings of this study indicate that a sufficient 25OHD intake can reduce physical symptoms at the onset of upper respiratory tract infection, particularly nasal symptoms, and may improve the quality of life at the time of onset.

## 1. Introduction

The common cold is a group of diseases that present with various upper respiratory symptoms, including nasal discharge, nasal obstruction, sneezing, sore throat, and cough. In the United States, it is estimated that children catch colds on average six to eight times a year, whereas adults are affected by colds two to four times a year, missing 22 million days of schooling, and 20 million days of work, respectively. Moreover, the economic loss due to colds is estimated to be US$25 billion per year [1,2].

The common cold is caused by a variety of viruses and bacteria, including rhinoviruses, coronaviruses, adenoviruses, RS virus, and parainfluenza viruses. Although vaccines may be effective in preventing the common cold, the multiple pathogenic viruses that cause these infections make complete prevention with vaccines difficult, and thus symptomatic treatment at the onset of the disease is the mainstay of treatment [3]. Moreover, even if a vaccine is developed, it may not produce neutralizing antibodies or antibody-dependent enhancement [4]. Therefore, in order to reduce the severity of the disease and to cure it at an early stage, it is important to control the onset of infection and reduce the attendant symptoms.

The intake of micronutrients such as vitamins A, C, and E, and minerals, such as zinc, stimulates the immune system by inducing epithelial barrier function and cellular immunity and promoting antibody production [5]. The effects of such micronutrients on the immune system indicate that they may prevent or reduce the severity of infectious diseases.

Vitamin D has been extensively studied in relation to respiratory tract infections and in the prevention and management of these infections. It has been reported that a reduction in the concentration of 25-hydroxyvitamin D in the blood is associated with an increased risk of respiratory tract infections [6,7,8], and it has also been found that vitamin D intake reduces the proportion of the incidence of respiratory tract infections to a greater than placebo intake [9]. In contrast, however, there have been conflicting reports questioning the treatment for respiratory tract infections with vitamin D [10]. Nevertheless, elevated levels of blood 25-hydroxyvitamin D may have some benefit in the prevention and management of these infections.

Vitamin D is synthesized by the skin through exposure to ultraviolet rays and is obtained from dietary sources, such fish, dairy products, and mushrooms, which contain vitamin D_2_ or D_3_. Regardless of whether it is synthesized or ingested, vitamin D is mainly hydroxylated at position 25 in the liver by CYP2R1 or CYP27A1, members of the cytochrome P450 superfamily of enzymes, to yield 25-hydroxyvitamin D [11].

While, 25-Hydroxyvitamin D binds to vitamin D-binding protein and circulates in the blood. On reaching the kidneys, 25-hydroxyvitamin D is subsequently hydroxylated at the 1α position by CYP27B1 to form 1α, 25 (OH)_2_ vitamin D [12,13], which is the active form of vitamin D that acts by binding to organs and tissues in which vitamin D receptors are expressed, as well as to immunocompetent cells [14,15,16]. CYP27B1 is also expressed in bronchial epithelial cells and is capable of hydroxylating 25-hydroxyvitamin D, thereby that indicating 1α, 25 (OH)_2_ vitamin D may play an important in the body’s initial defense against pathogens [17].

We have previously studied the effects of 25-hydroxyvitamin D_3_ on upper respiratory tract infection (URTI) in Japanese subjects with insufficient or deficient 25-hydroxyvitamin D levels in the blood, and the results indicated that 25-hydroxyvitamin D_3_ is effective in improving overall symptoms, and quality of life of subjects with URTI and shortening the duration of the URTI [18]. However, it has yet to be determined which types and symptoms of URTI are affected by 25-hydroxyvitamin D_3_ intake, nor has it been established what effects it might have on the efficacy of other medications.

In this study, we conducted a post hoc analysis of data obtained from our previous clinical trials to evaluate the effects of 25-hydroxyvitamin D_3_ intake on the number of symptoms and events at the onset of URTI and the proportion of medication (antibiotics and antipyretics) use.

## 2. Materials and Methods

### 2.1. Study Cohort

We performed a post hoc analysis using data obtained from a previous clinical trial [18]. The study was a prospective, randomized, double-blind, placebo-controlled, parallel-group comparison study, in which Japanese men and women with insufficient or deficient blood levels (≤75 nmol/L) of 25-hydroxyvitamin D received 10 µg of 25-hydroxyvitamin D_3_ (25OHD) per day. Double-blind indicates that randomization to the placebo or 25OHD group was blinded to all relevant parties, including subjects, researchers, and physicians. Details of the characteristics of the subjects and the inclusion and exclusion criteria can be found in the original paper [18]. In this study, 215 subjects in the per protocol set (PPS) were included in the post hoc analysis. Raw data, such as severity score, medication, and laboratory test values were obtained from the corresponding author (Y.S).

Subjects completed a Wisconsin Upper Respiratory Symptom Survey-21 (WURSS-21) and a diary of changes in physical conditions and medication status during the test food intake.

### 2.2. Study Implementation

In this study, physical examination and clinical tests were conducted at the community hospital clinics, and a system was established for the management of subjects and the implementation of the study. The investigator responsible of all aspects of the study, gave instructions to the subjects, provided necessary explanations, obtained their written informed consent, interviewed them, checked for and assessed adverse events, and managed the study system. The original study was conducted from December 2015 to September 2016. 

### 2.3. Ethical Statement

The original study was conducted having initially been reviewed and approved by the HUMA R&D Ethical Review Committee, which consisted of third parties who were not involved in the study (approval date: 11 December 2015). The study was conducted in accordance with the Declaration of Helsinki (adopted in June 1964 and revised in October 2013) and the Ethical Guidelines for Medical Research Involving Human Subjects (Ministry of Education, Culture, Sports, Science and Technology and Ministry of Health, Labour and Welfare Notification No. 3 of 2014). The content of the study was registered in UMIN Clinical Trials Registry (https://www.umin.ac.jp/ctr/) prior to commencement. The purpose and details of the study were fully explained to the subjects by the investigators, and their voluntary consent was obtained in writing. 

### 2.4. Trial Supplement

A hard capsule formulation containing 10 μg of 25OHD was used. The placebo was prepared by replacing 25OHD with crystalline cellulose, such that the color and other characteristics were indistinguishable from those of the 25OHD capsules. While, 25OHD was provided free of charge by DSM Nutritional Products, Ltd. (Heanor, UK).

### 2.5. Upper Respiratory Tract Symptom Survey

The Japanese version of the WURSS-21 was used to assess the incidence and severity of physical symptoms of upper respiratory infections, as well as subject quality of life [19]. Questions (0 to 7 on a Likert scale: 0 = no symptoms, 1 = somewhat sick, 3 = slightly sick, 5 = moderately sick, 7 = very sick) were answered daily during the study period, and were used to determine the onset and duration of the disease. The second to the eleventh 10 questions of the WURSS-21 relate to 10 physical symptoms of URTI (Runny nose, Plugged nose, Sneezing, Sore throat, Scratchy throat, Cough, hoarseness, Head congestion, Chest congestion, and Feeling tired) on a Likert scale of 0 to 7 (0 = none, 1 = very mild, 3 = mild, 5 = moderate, 7 = severe), and the sum of these scores at the time of URTI onset was used for evaluation. The subsequent nine questions (12th to 20th) concern nine quality of life events (Think clearly, Sleep well, Breathe easily, Walk, climb stairs, exercise, accomplish daily activities, work outside the home, work inside the home, interact with others, and live your personal life), the sum of the scores of which reflect the quality of life at the time of URTI onset.

The date of URTI onset was set to the date when the subject selected a score values from 1 to 7 after selecting 0 for the first two consecutive days, and the date of disappearance was set to the date 2 days before (or the date when the subject selected 1 to 7 for the final time) the subjects selected 0 for the second consecutive day after the onset. In this study, URTI events were defined as those with a duration of illness (the period from the date of onset to the date of resolution) of more than 2 days and a maximum physical severity score of more than 10 during the period of illness. 

### 2.6. Outcome Measures

As outcome measures, we used the following: The WURSS-21 question scores and proportion of incidence of each symptom, the number of events during the period of URTI, the proportion of antibiotic and antipyretic analgesic (acetaminophen and NSAIDs) use, and the proportion of medication use at the time of URTI. The symptom score and proportion of incidence rate, as well as the proportion of medication use at the time of URTI onset, were evaluated only in subjects with URTIs. 

According to the task team guidelines described by Holick et al., the risk of osteomalacia and rickets is increased in a deficiency state where the serum 25-hydroxyvitamin D level is 50 nmol/L (= 20 ng/mL) or lower, and that the state where the serum 25-hydroxyvitamin D level is between 50 nmol/mL and 75 nmol/L (= 30 ng/mL) is an insufficiency state where the risk of falling is increased [20,21]. We conducted a subgroup analysis according to the sufficiency of 25-hydroxyvitamin D in the blood at baseline. The serum 25-hydroxyvitamin D levels were measured using the 25-hydroxy-vitamin D 125I RIA Kit (DiaSorin S. P. A, Saluggia, Italy) at SRL Inc. (Tokyo, Japan) in the previous study [18].

### 2.7. Statistical Analysis

Student’s *t* test was used to assess the WURSS-21 score of each subjects. The proportion of incidence of each symptom and the proportion of drug use were assessed using Fisher’s exact test, and the Mann–Whitney U test was used to assess the number of URTI events. Due to the exploratory nature of this study and the small number of subjects analyzed, 10% on both sides was considered marginally significant, in order not to overlook the efficacy of 25OHD.

All analyses were performed using JMP^®^ 14.1.0 (SAS Institute Inc., Cary, NC, USA). Owing to the exploratory nature of this analysis, we did not adjust for the multiplicity of the tests.

## 3. Results

### 3.1. Background Information on the Study Subjects

Background information pertaining to the subjects participating in this study is presented in Table 1 and Table 2. 

Overall, there was a significant gender difference between the 25OHD and placebo groups, with a larger number of males and fewer females in the 25OHD group than in the placebo group. Otherwise, there were no significant differences between the groups.

Among the PPS, 43 subjects in the placebo group and 41 in the 25OHD group developed URTIs. In the 25-hydroxyvitamin D-insufficient group, 17 subjects in the placebo group and 15 in the 25OHD group developed URTIs, whereas 26 subjects in the 25-hydroxyvitamin D-deficient group and 26 in the 25OHD group developed URTIs. 

### 3.2. Severity of Upper Respiratory Tract Infection Symptoms

The scores for physical symptoms and quality of life, and the number of subjects with each symptom, are shown in Table 3 and Table 4. The 25OHD group had lower levels of all symptoms than the placebo group: “Runny nose,” “Sneezing,” and “Head congestion” were significantly lower in the 25OHD group, whereas “Plugged nose,” “Sore throat,” “Scratchy throat,” and “Cough” were marginally significantly lower (Table 3). These results indicate that 25OHD intake may alleviate the symptoms of URTI in general and nasal symptoms in particular. However, there were no significant differences between the two groups with respect to the incidence of each symptom (Table 3).

With the exception of “Breathe easily,” all quality of life-related items were significantly lower in the 25OHD group than in the placebo group (Table 4). We speculate that 25OHD intake improved the quality of life by reducing the symptoms, mainly nasal symptoms, at the onset of URTI. The proportion of incidence of “Breathe easily” was marginally significantly lower in the 25OHD group than in the placebo group (Table 4).

In the 25-hydroxyvitamin D-insufficient group, individuals in the 25OHD group had marginally significantly lower levels of “Plugged nose,” “Sneezing,” and “Head congestion” than those in the placebo group (Table 3), whereas in the 25-hydroxyvitamin D-deficiency group, those in the 25OHD group had marginally significantly lower levels of “Runny nose,” “Sore throat,” and “Cough” (Table 3). With the exception of “Breathe easily,” all quality of life measures were significantly lower in the 25OHD group than in the placebo group (Table 4). In the 25-hydroxyvitamin D deficiency group, among the proportion of incidence of physical severity items, the “Cough” category was significantly lower (Table 3). We accordingly hypothesized that improvements in the quality of life-related parameters associated with 25OHD intake were related to a reduction in physical symptoms.

The scores of the 25-hydroxyvitamin D-deficient group were generally higher than those of the 25-hydroxyvitamin D-insufficient group, thereby indicating that a deficiency of 25-hydroxyvitamin D in the blood tended to be associated with URTI of greater severity. In addition, 25OHD supplementation in the 25-hydroxyvitamin D-deficient group was considered to be effective. Given that each score for the 25OHD-deficient group approached that recorded for the 25-hydroxyvitamin D-insufficient group, we take this to be indicative of an effect of 25OHD supplementation.

### 3.3. Number of Upper Respiratory Tract Infection Events

A summary of the number of URTI events identified during the study period is shown in Table 5. The total numbers of URTI events for the placebo and 25OHD groups were 66, and 52, respectively. The difference in the number of URTI between the two groups was the largest and was marginally significant (Placebo group, 17 times; 25OHD group, 7 times). With respect to the difference in serum 25-hydroxyvitamin D sufficiency, we found that the 25-hydroxyvitamin D-deficient group was characterized by a higher number of URTI events, with 14 events in the placebo group versus five in the 25OHD group at 9 to 12 weeks. These results indicate that the URTIs identified in the same subjects may have been suppressed by 25OHD intake, and we accordingly speculate that continuous intake of 25OHD would inhibit the development of URTIs.

### 3.4. Proportion of Medication Use

Table 6 shows the proportions of antibiotics and antipyretic analgesics (acetaminophen and NSAIDs) used by study participants, respectively. Over the course of the study, we detected no significant difference between the placebo and 25OHD groups with respect to the proportion of antibiotic use. Similarly, there were no differences between the two groups regarding the proportion of antibiotics used to treat URTI (Table 6). Likewise, we found no differences between the placebo and 25OHD groups in terms of antipyretic analgesia usage, nor were there any significant differences in the rate of use for dealing with URTI. However, the use of acetaminophen for URTI was observed to be higher in the 25OHD group than in the placebo group, whereas the use of NSAIDs was lower in the 25OHD group, although the difference was not significant.

For both the 25-hydroxyvitamin D-insufficient and -deficient groups, there were no significant differences between the 25OHD and placebo groups with respect to the proportions of antibiotic and antipyretic analgesic usage. In contrast, in the 25-hydroxyvitamin D-insufficient group, there was a trend toward a higher proportion of acetaminophen use for URTI in the 25OHD group, which was higher than that observed in the 25-hydroxyvitamin D-deficient group. The proportion of NSAIDs use of 25OHD was lower than that of the placebo group, thus indicating an anti-inflammatory effect of 25OHD intake. There was also a trend for higher acetaminophen use in individuals receiving 25OHD in the 25-hydroxyvitamin D-insufficient group.

In Japan, numerous common cold medications available in pharmacies contain acetaminophen, and given that the severity of URTI was lower in the 25OHD group than in the placebo group, more subjects in the former group used over-the-counter cold medications, and accordingly, the proportion of acetaminophen use in the 25OHD group was higher than that in the placebo group.

## 4. Discussion

Given that respiratory tract infections are caused by a diverse range of viruses and bacteria, it has been difficult to develop effective treatments for such infections. A number of studies have been conducted to investigate the preventive and control effects of vitamin D and other micronutrients on respiratory tract infections [5], among which is 25OHD, a metabolite of vitamin D. In this study, we conducted a post hoc analysis using data from a previous study that examined the effects of 25OHD intake.

The results indicated that 25OHD intake can generally alleviate various symptoms of URTI, such as runny nose, sneezing, and head congestion, and can also reduce the incidence of URTI events and the proportion of NSAID usage. Both these effects can be attributed to an increase in 25-hydroxyvitamin D concentrations in the blood following the intake of 25OHD. Indeed, we found that individuals in the 25-hydroxyvitamin D-deficient group had a higher severity score for each symptom at the onset of URTI, thereby indicating that lower blood levels of 25-hydroxyvitamin D may be correlated with the severity of URTI, and that an increase in blood 25-hydroxyvitamin D following 25OHD intake contributes to reducing disease severity.

It is conceivable that these reductions in the severity of URTI in response to 25OHD intake are associated with a reduction in the levels of cytokines and chemokines, which have been investigated with respect to vitamin D metabolism, modulation of the renin-angiotensin system, regulation of neutrophil activity, maintenance of pulmonary epithelial barrier function, and promotion of epithelial repair [22,23,24]. A majority of the studies that have examined vitamin D functionality in vitro have focused on 25OHD using hydroxylated 1α, 25 (OH)_2_ vitamin D. It is assumed that most of the functions of vitamin D are vitamin D receptor mediated. Both the affinity of 25OHD for vitamin D receptors and its concentration in blood are approximately 1,000 times greater than those of 1α, 25 (OH)_2_ vitamin D, indicating that 25OHD may also directly exert a variety of functional effects.

Recently, a deficiency in vitamin D has been reported to be associated with an increased risk of COVID-19 infection, and clinical trials on the efficacy of vitamin D and 25OHD for COVID-19 have been conducted and are underway (NCT04334005, NCT04363840, NCT04335084, NCT04351490, NCT04366908, NCT04386850) [25]. It has also been reported that 25OHD administration, although limited efficacy, inhibits the severity of COVID-19 [26]. If indeed vitamin D and 25OHD intake is shown to be effective against COVID-19 and other respiratory tract infections, the use of these micronutrients may become a widespread, inexpensive, and simple countermeasure for the control of respiratory tract infections.

## 5. Conclusions

The findings of this study indicate that 25OHD intake in individuals with both insufficient and deficient levels of 25-hydroxyvitamin D in the blood can be effective in reducing the severity of nasal and other symptoms at the onset of URTI, and also improve the associated quality of life. However, owing to the small number of cases assessed in the present study, our findings should be interpreted with caution.

## Figures and Tables

**Table 1:** Demographic characteristics of the efficacy analysis set.

		Placebo	25OHD	*p* Value *
		(*n* = 105)	(*n* = 110)	
Age	(years)	52.6 ± 6.7	52.8 ± 6.2	0.788
Gender	(male/female)	25/80	41/69	0.039
Body mass index	(kg/m^2^)	21.2 ± 1.6	21.3±1.6	0.729
Serum 25-hydroxyvitamin D	(nmol/L)	48.6 ± 13.1	49.1 ± 13.8	0.798
Serum 1α, 25 (OH)_2_ D	(pg/mL)	54.6 ± 14.7	53.3 ± 16.6	0.539
Intact PTH	(pg/mL)	50.6 ± 14.7	47.8 ± 15.1	0.163
Serum calcium	(mg/dL)	9.29 ± 0.33	9.31 ± 0.33	0.722
Urinary calcium	(mg/mg creatinine)	0.112 ± 0.079	0.105 ± 0.070	0.496

Abbreviation: Intact PTH (Intact parathyroid hormone). Values are represented as the mean ± SD. Placebo and 25OHD denote the placebo and 25-hydroxivitamin D_3_ group, respectively. * Between group comparisons were assessed using Student’s *t* test or Fisher’s exact test.

**Table 2:** Demographic characteristic of the 25-hydorxyvitamin D insufficiency and deficiency groups.

	Serum 25-Hydroxyvitamin D Levels					
	Insufficiency			Deficiency		
	Placebo	25OHD	*p* Value *	Placebo	25OHD	*p* Value *
	(*n* = 45)	(*n* = 49)		(*n* = 60)	(*n* = 61)	
Age (years)	54.0 ± 7.6	53.7 ± 7.0	0.850	51.5 ± 5.8	52.1 ± 5.4	0.570
Gender (male/female)	12/33	23/26	0.055	13/47	18/43	0.406
Body mass index (kg/m^2^)	21.1 ± 1.7	21.3 ± 1.6	0.461	21.3 ± 1.6	21.3 ± 1.6	0.890
Serum 25 hydroxyvitamin D (nmol/L)	61.4 ± 6.6	62.2 ± 7.8	0.618	39.0 ± 7.1	38.6 ± 6.8	0.731
Serum 1α, 25 (OH)_2_ D (pg/mL)	58.8 ± 17.5	57.8 ± 19.1	0.794	51.5 ± 11.4	49.7 ± 13.3	0.426
Intact PTH (pg/mL)	48.6 ± 12.9	43.8 ± 11.5	0.056	52.1 ± 15.9	51.0 ± 16.8	0.704
Serum calcium (mg/dL)	9.29 ± 0.31	9.30 ± 0.34	0.919	9.29 ± 0.34	9.31 ± 0.32	0.701
Urinary calcium (mg/mg creatinine)	0.114 ± 0.064	0.111 ± 0.085	0.859	0.110 ± 0.089	0.100 ± 0.056	0.443
Creatinine clearance (mL/min)	90.0 ± 17.1	91.4 ± 14.6	0.660	90.8 ± 14.7	97.0 ± 25.1	0.100

Values are represented as the mean ± SD. Placebo and 25OHD denote the placebo and 25-hydroxivitamin D_3_ group, respectively. * Between group comparisons were assessed using Student’s *t* test or Fisher’s exact test.

**Table 3:** Effect of 25-hydroxyvitamin D_3_ on physical the severity of upper respiratory tract infection.

	Serum 25-Hydroxyvitamin D Levels								
	Overall			Insufficiency			Deficiency		
Items	Placebo (*n* = 43)	25OHD (*n* = 41)	*p* Value *	Placebo (*n* = 17)	25OHD (*n* = 15)	*p* Value *	Placebo (*n* = 26)	25OHD (*n* = 26)	*p* Value *
Runny nose	45.7 ± 55.6	23.1 ± 33.1	0.027	36.2 ± 47.0	15.6 ± 13.3	0.111	51.8 ± 60.6	27.4 ± 40.0	0.092
	(42/43)	(40/41)	(1.000)	(17/17)	(14/15)	(0.468)	(25/26)	(26/26)	(1.000)
Plugged nose	36.5 ± 48.1	19.9 ± 35.4	0.077	31.6 ± 44.1	10.3 ± 13.3	0.081	39.6 ± 51.2	25.5 ± 42.6	0.283
	(41/43)	(37/41)	(0.427)	(16/17)	(13/15)	(0.588)	(25/26)	(24/26)	(1.000)
Sneezing	27.5 ± 41.4	12.2 ± 16.0	0.030	23.0 ± 27.8	8.1 ± 6.9	0.052	30.4 ± 48.6	14.6 ± 19.2	0.129
	(39/43)	(36/41)	(0.735)	(15/17)	(12/15)	(0.645)	(24/26)	(24/26)	(1.000)
Sore throat	35.4 ± 52.6	20.0 ± 17.8	0.078	22.5 ± 19.4	19.5 ± 14.7	0.628	43.8 ± 65.0	20.3 ± 19.7	0.082
	(41/43)	(38/41)	(0.672)	(16/17)	(14/15)	(1.000)	(25/26)	(24/26)	(1.000)
Scratchy throat	43.6 ± 57.1	25.0 ± 36.7	0.081	35.4 ± 40.1	21.7 ± 21.8	0.249	48.9 ± 66.2	26.8 ± 43.3	0.161
	(42/43)	(39/41)	(0.611)	(16/17)	(14/15)	(1.000)	(26/26)	(25/26)	(1.000)
Cough.	40.6 ± 60.1	20.8 ± 25.1	0.053	48.2 ± 78.9	27.7 ± 21.7	0.338	35.7 ± 44.9	16.8 ± 26.4	0.070
	(39/43)	(32/41)	(0.137)	(14/17)	(14/15)	(0.602)	(25/26)	(18/26)	(0.023)
Hoarseness	28.3 ± 51.4	16.5 ± 15.5	0.163	30.5 ± 61.8	16.9 ± 13.0	0.408	26.8 ± 44.7	16.3 ± 17.0	0.268
	(39/43)	(37/41)	(1.000)	(16/17)	(15/15)	(1.000)	(23/26)	(22/26)	(1.000)
Head congestion	30.6 ± 50.6	12.6 ± 19.3	0.035	25.1 ± 37.0	7.9 ± 9.8	0.091	34.2 ± 58.3	15.2 ± 22.8	0.129
	(37/43)	(32/41)	(0.400)	(14/17)	(9/15)	(0.243)	(23/26)	(23/26)	(1.000)
Chest congestion	30.1 ± 48.1	16.6 ± 33.1	0.139	26.6 ± 46.7	13.8 ± 17.9	0.325	32.3 ± 49.8	18.2 ± 39.6	0.261
	(36/43)	(34/41)	(1.000)	(15/17)	(13/15)	(1.000)	(21/26)	(21/26)	(1.000)
Feeling tired	53.5 ± 77.5	34.6 ± 55.6	0.204	41.1 ± 63.7	33.2 ± 56.0	0.715	61.6 ± 85.5	35.3 ± 56.5	0.197
	(43/43)	(41/41)	(-)	(17/17)	(15/15)	(-)	(26/26)	(26/26)	(-)

Values are represented as the mean ± SE. Placebo and 25OHD denote the placebo and 25-hydroxivitamin D_3_ groups, respectively. Numbers in parentheses indicate the proportion of subjects with upper respiratory tract infection and each symptom. * Between group comparisons were assessed using Student’s *t* test or Fisher’s exact test. Values in parentheses are *p* values for comparison of the proportion of each symptom.

**Table 4:** Effect of 25-hydroxyvitamin D_3_ on the mental severity (QOL) of upper respiratory tract infection.

	Serum 25-hydroxyvitamin D Levels								
	Overall			Insufficiency			Deficiency		
Items	Placebo (*n* = 43)	25OHD (*n* = 41)	*p* Value *	Placebo (*n* = 17)	25OHD (*n* = 15)	*p* Value *	Placebo (*n* = 26)	25OHD (*n* = 26)	*p* Value *
Think clearly	33.4 ± 51.9	13.7 ± 12.3	0.019	26.1 ± 46.1	15.5 ± 14.2	0.397	38.2 ± 55.7	12.7 ± 11.3	0.026
	(39/43)	(36/41)	(0.735)	(14/17)	(13/15)	(1.000)	(25/26)	(23/26)	(0.609)
Sleep well	29.7 ± 52.0	12.0 ± 14.0	0.038	23.6 ± 49.3	15.7 ± 13.8	0.552	33.7 ± 54.3	9.9 ± 13.9	0.035
	(40/43)	(34/41)	(0.189)	(15/17)	(11/15)	(0.382)	(25/26)	(23/26)	(0.609)
Breathe easily	29.2 ± 48.5	13.8 ± 21.0	0.065	23.4 ± 42.2	11.7 ± 10.2	0.304	33.0 ± 52.7	15.0 ± 25.4	0.123
	(41/43)	(34/41)	(0.084)	(16/17)	(11/15)	(0.160)	(25/26)	(23/26)	(0.609)
Walk, climb stairs, exercise	28.6 ± 48.0	10.7 ± 12.9	0.023	22.2 ± 42.1	10.8 ± 15.0	0.330	32.8 ± 51.8	10.6 ± 11.8	0.038
	(38/43)	(33/41)	(0.375)	(14/17)	(10/15)	(0.423)	(24/26)	(23/26)	(1.000)
Accomplish daily activities	31.4 ± 47.1	13.1 ± 12.2	0.018	24.2 ± 42.0	15.3 ± 15.0	0.442	36.0 ± 50.4	11.9 ± 10.4	0.020
	(40/43)	(35/41)	(0.307)	(15/17)	(12/15)	(0.645)	(25/26)	(23/26)	(0.609)
Work outside the home	34.1 ± 50.2	14.7 ± 13.5	0.019	23.4 ± 42.4	18.3 ± 18.0	0.669	41.1 ± 54.4	12.6 ± 9.8	0.011
	(41/43)	(35/41)	(0.151)	(16/17)	(13/15)	(0.588)	(25/26)	(22/26)	(0.349)
Work inside the home	30.1 ± 46.6	12.0 ± 11.8	0.017	23.5 ± 42.1	14.1 ± 14.8	0.418	34.4 ± 49.6	10.7 ± 9.8	0.020
	(40/43)	(35/41)	(0.307)	(16/17)	(12/15)	(0.319)	(24/26)	(23/26)	(1.000)
Interact with others	34.7 ± 49.2	14.8 ± 14.5	0.014	27.6 ± 45.9	19.4 ± 19.0	0.521	39.3 ± 51.7	12.1 ± 10.6	0.011
	(39/43)	(34/41)	(0.345)	(15/17)	(13/15)	(1.000)	(24/26)	(21/26)	(0.418)
Live your personal life	38.2 ± 49.4	17.2 ± 16.7	0.011	32.9 ± 46.5	19.9 ± 19.7	0.324	41.7 ± 51.8	15.7 ± 14.9	0.017
	(41/43)	(38/41)	(0.672)	(16/17)	(14/15)	(1.000)	(25/26)	(24/26)	(1.000)

Values are represented as the mean ± SE. Placebo and 25OHD denote the placebo and 25-hydroxivitamin D_3_ groups, respectively. Numbers in parentheses indicate the proportion of subjects with upper respiratory tract infection and each symptom. * Between group comparisons were assessed using Student’s *t* test or Fisher’s exact test. Values in parentheses are *p* values for comparison of the proportions of each symptom.

**Table 5:** The number of upper respiratory tract infection (URTI) episodes after commencing supplement intake.

				Time after Start of Intake (Weeks)			
Serum 25-Hydroxyvitamin D Levels	Group		Total	1–4	5–8	9–12	13–16
Overall	Placebo	No. of URTI events	66	24	19	17	6
		No. of subjects with URTI onset	43	21	19	14	6
	25OHD	No. of URTI events	52	24	15	7	6
		No. of subjects with URTI onset	41	22	14	7	6
		*p* value *	0.450	0.971	0.292	0.078	0.936
Insufficiency	Placebo	No. of URTI events	23	9	10	3	1
		No. of subjects with URTI onset	17	8	10	3	1
	25OHD	No. of URTI events	19	10	5	2	2
		No. of subjects with URTI onset	15	10	5	2	2
		*p* value *	0.446	0.794	0.115	0.585	0.619
Deficiency	Placebo	No. of URTI events	43	15	9	14	5
		No. of subjects with URTI onset	26	13	9	11	5
	25OHD	No. of URTI events	33	14	10	5	4
		No. of subjects with URTI onset	26	12	9	5	4
		*p* value *	0.740	0.796	1.000	0.089	0.715

Placebo and 25OHD denote the placebo and 25-hydroxivitamin D_3_ groups, respectively. * Between group comparisons were assessed using the Mann–Whitney U test.

**Table 6:** The proportions of medicine used.

Serum 25-Hydoroxyvitamin D Levels		Antimicrobials		Antipyretics Analgesic		Acetaminophen		NSAIDs	
		Total	for URTI	Total	for URTI	Total	for URTI	Total	for URTI
Overall	Placebo	11/105	5/43	25/105	10/43	10/105	6/43	21/105	8/43
	(%)	(10.5)	(11.6)	(23.8)	(23.3)	(9.5)	(14.0)	(20.0)	(18.6)
	25OHD	9/110	3/41	24/110	12/41	13/110	11/41	15/110	3/41
	(%)	(8.2)	(7.3)	(21.8)	(29.3)	(11.8)	(26.8)	(13.6)	(7.3)
	*p* value *	0.642	0.713	0.748	0.623	0.662	0.179	0.273	0.196
Insufficiency	Placebo	2/45	1/17	8/45	3/17	2/45	1/17	6/45	2/17
	(%)	(4.4)	(5.9)	(17.8)	(17.6)	(4.4)	(5.9)	(13.3)	(11.8)
	25OHD	5/49	2/15	10/49	5/15	5/49	5/15	7/49	1/15
	(%)	(10.2)	(13.3)	(20.4)	(33.3)	(10.2)	(33.3)	(14.3)	(6.7)
	*p* value *	0.438	0.589	0.798	0.424	0.438	0.076	1.000	1.000
Deficiency	Placebo	9/60	4/26	17/60	7/26	8/60	5/26	15/60	6/26
	(%)	(15.0)	(15.4)	(28.3)	(26.9)	(13.3)	(19.2)	(25.0)	(23.1)
	25OHD	4/61	1/26	14/61	7/26	8/61	6/26	8/61	2/26
	(%)	(6.6)	(3.8)	(23.0)	(26.9)	(13.1)	(23.1)	(13.1)	(7.7)
	*p* value *	0.154	0.350	0.537	1.000	1.000	1.000	0.110	0.249

Placebo and 25OHD denote the placebo and 25-hydroxivitamin D_3_ group, respectively. * Between group comparisons were assessed using Fisher’s exact test.
